# Scaled-up non-thermal plasma-generating device Plasmatico enables effective and harmless disinfection of personal protective equipment

**DOI:** 10.1038/s41598-025-19520-7

**Published:** 2025-10-13

**Authors:** Eva Vaňková, Klára Obrová, Jan Hodek, Anna Machková, Petra Kašparová, Josef Khun, Eliška Lokajová, Lucie Ulrychová, Paola Fürhacker, Michal Sláma, Radek Přikryl, Lukáš Kalina, Štěpán Krobot, Thomas Lion, Jan Weber, Vladimír Scholtz

**Affiliations:** 1 Department of Physics and Measurements, University of Chemistry and Technology, Prague, Czech Republic; 2 Department of Biotechnology, University of Chemistry and Technology, Prague, Czech Republic; 3 St. Anna Children’s Cancer Research Institute, Vienna, Austria; 4 Institute of Organic Chemistry and Biochemistry of the Czech Academy of Sciences, Prague, Czech Republic; 5 Faculty of Science, University of Hradec Kralove, Hradec Králové, Czech Republic; 6 Faculty of Chemistry, Brno University of Technology, Brno, Czech Republic; 7 Department of Pediatrics, Medical University of Vienna, Vienna, Austria

**Keywords:** Cold atmospheric plasma, Disinfection, MRSA, SARS-CoV-2, Virus inactivation

## Abstract

The COVID-19 pandemic highlighted the urgent need to find effective methods for disinfecting items such as personal protective equipment (PPE) in case a similar situation occurs in the future. To address this problem, we selected FFP2 masks, a common PPE that cannot be easily disinfected, and developed a scaled-up non-thermal plasma (NTP)-generating device Plasmatico v1.0, which was constructed as part of a series of prototypes. It is equipped with multiple electrodes within a large exposure chamber. Using Plasmatico v1.0, we achieved complete disinfection of FFP2 masks contaminated with SARS-CoV-2, influenza A virus and human rhinovirus after 90 min, while highly resistant human adenovirus was inactivated after 120 min. In addition, *Pseudomonas aeruginosa* and methicillin-resistant *Staphylococcus aureus* (MRSA) were disinfected within 90 min. Furthermore, the mechanical and surface properties of FFP2 masks remained largely unaffected even after extended NTP exposure (up to 24 h), as shown using tensile test and surface analysis. The newly developed Plasmatico v1.0 device represents an effective, harmless, and affordable solution for disinfecting daily used objects such as FFP2 masks and highlights the potential of NTP technology for widespread application in public health, including improved preparedness for potential future pandemics and for keeping infection outbreaks under control.

**Supplementary Information:**

The online version contains supplementary material available at 10.1038/s41598-025-19520-7.

## Introduction

The COVID-19 pandemic, which caught the world largely unprepared, prompted a rapid development of novel or improved tools for disinfecting or decontaminating everyday objects, including sensitive materials such as personal protective equipment (PPE) (e.g., face masks). These disinfection methods have to ensure that the protective efficacy of the masks against airborne pathogens is not affected^1–3^. It is not just a matter of dealing with a particular pandemic, but rather the need to be better prepared for any situation of this type. One of the fields actively involved in addressing this problem is plasma physics, as the use of non-thermal plasma (NTP) as a disinfection technique is very effective and gentle to the surface to be treated^4^. NTP is an ionized gas of low temperature, containing reactive oxygen and nitrogen species (RONS), charged particles, photons of various wavelengths and electromagnetic fields. There are many options in generating NTP, which largely determine its final properties. The most common way is gas ionization by electrical discharges with various electrical characteristics (e.g., sinusoidal, pulsed, direct current—DC, alternating current—AC). In terms of electrode configuration, the most common types of discharges are corona discharges and dielectric barrier discharges (DBD)^5^.

NTP is a promising technology for the field of medicine with an increasing number of applications in various therapeutic areas. Among others, it has high potential in promoting cell growth and stimulating tissue repair, and is also being explored for the treatment of cancer through selective cell apoptosis^6–8^. Recent studies highlight its efficacy in improving wound healing, particularly in chronic and infected wounds, and its ability to disrupt bacterial biofilms^9^ without damaging surrounding tissues^10^. NTP is already being used in dermatology for the treatment of acne^11^, as well as in dentistry, e.g., for biofilm removal^12^ or disinfection of dental root canals^13^.

The interest in new antiviral strategies considerably increased upon the COVID-19 pandemic, triggering active discussion on NTP use for virus inactivation^4,14^. The control of viral transmission via contact with contaminated materials is a major challenge, especially in the hospital environment. It has been shown that NTP can effectively inactivate SARS-CoV-2 and other respiratory viruses on non-porous and porous surfaces commonly found in healthcare facilities and is therefore a very promising tool for virus elimination^15–17^.

A great variety of NTP-generating devices effective against important pathogens, including the ESKAPE^18^, have been developed but most of them remain only at laboratory prototype stage. However, a successful scale-up of NTP-generating prototypes to widely applicable devices is possible and has already been performed in various fields^19,20^.

In this study, we sequentially developed three DC-based NTP-generating devices and tested them for the disinfection of FFP2 face masks. We started with Laboratory Prototype 1 equipped with two corona discharges based on a point-to-ring electrode system, subsequently we increased the chamber volume and used four discharges in Laboratory Prototype 2 and finally we developed the Plasmatico v1.0 with the largest chamber volume and eight discharges, with wide applicability while maintaining the same efficiency and affordability. We tested the inhibitory efficacy of these devices using four important virus species (Severe Acute Respiratory Syndrome Coronavirus 2—SARS-CoV-2, influenza A virus—IAV, human rhinovirus—HRV and human adenovirus—HAdV) as well as two common bacterial pathogens (*Pseudomonas aeruginosa* and methicillin-resistant *Staphylococcus aureus—*MRSA), which belong to ESKAPE group. Simultaneously, we evaluated in detail the effect of Plasmatico v1.0 on the FFP2 face mask material with respect to any potential damage caused by the exposure. We conclude that Plasmatico v1.0 is an effective, gentle, affordable, easily portable device, ready for use in almost any conditions, with a common electrical plug being the only resource needed. In this way, we successfully translated our basic research project into practical application of NTP, as Plasmatico v1.0 is readily suitable for the disinfection of everyday objects.

## Results

### Development of scaled-up NTP-generating devices with multiple point-to-ring electrode systems

**Fig. 1:**
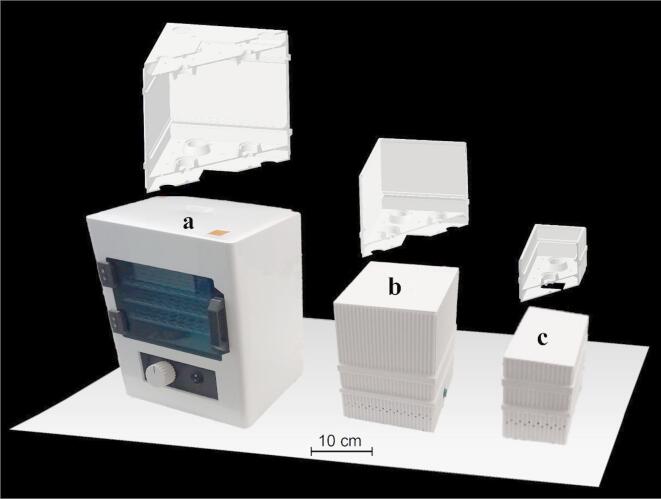
Schematic view and photographs of scaled-up NTP generating devices. Three devices (Plasmatico v1.0—**a**, Laboratory prototype 2—**b** and Laboratory prototype 1—**c**) were sequentially developed to achieve a chamber volume allowing the insertion of daily used items while maintaining sufficient disinfection efficiency. Design changes and an increasing number of point-to-ring electrode systems generating NTP are depicted.

In order to adapt the previously developed NTP device^21,22^ for disinfection of items used daily, it was necessary to scale-up the exposure chamber. To develop a device with the sufficient chamber volume to fit larger objects, while maintaining disinfection efficiency, three devices with multiple point-to-ring electrode systems were sequentially developed (schematic views with scale bar are shown in Fig. 1): Laboratory prototype 1 with two electrode systems and a chamber of 1560 cm^3^, Laboratory prototype 2 with four electrode systems and twice as big cubic chamber (3120 cm^3^), and Plasmatico v1.0, the prototype aimed for commercial use, with eight electrode systems and a cubic chamber of 5440 cm^3^.

### Determination of NTP virucidal efficiency in different locations of Laboratory prototype 1 chamber

To address the impact of sample placement within the device chamber on disinfection efficiency, we selected four different locations within the Laboratory prototype 1 and used HRV samples for testing (Supplementary Fig. S1). Three samples were facing towards the bottom of the chamber, where the NTP source is placed: either directly above the source (position 1), distant from the source (position 2), or glued inside a Petri dish (position 4). The last sample was facing upwards, away from the NTP source (position 3, in a Petri dish). It was clearly demonstrated that after 30 min of NTP exposure, HRV was inhibited to a greater degree when contamination faced the NTP source (positions 1, 2 and 4) than in the position 3 facing away from the NTP source (Supplementary Fig. S2). Complete inactivation was not achieved in any of the tested positions after 30 min of NTP exposure. The strongest inactivation (less than 1 log_10_ of residual infectious virus) was achieved in position 1, while the lowest inactivation was achieved in position 3 (2.7 log_10_ of residual infectious virus). Position 3 was thus used in subsequent experiments with all scaled-up devices, ensuring unbiased and stringent efficiency testing.

### Virucidal activity of NTP-generating devices against SARS-CoV-2, IAV, HRV and HAdV

**Fig. 2:**
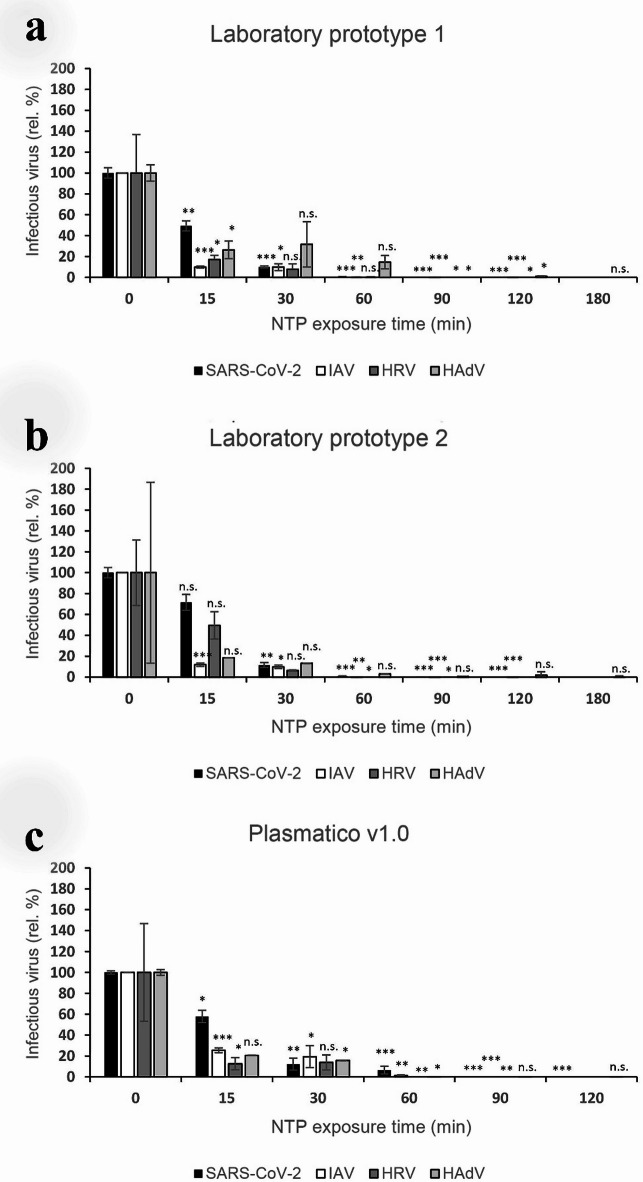
Virucidal activity of three NTP-generating devices tested using Severe Acute Respiratory Syndrome Coronavirus 2 (SARS-CoV-2), influenza A virus (IAV), human rhinovirus (HRV) and human adenovirus (HAdV). (**a**) Laboratory prototype 1, (**b**) Laboratory prototype 2, (**c**) Plasmatico v1.0. NTP exposure was performed after contamination of parafilm squares by virus suspensions and their insertion into each device. Results are normalized to controls and expressed as relative percentages of infectious virus (rel. %) ± standard error of mean. The log_10_ TCID_50_ decreases are shown in Supplementary Table S1. ***:*p* < 0.0005- , **:*p* < 0.005—, *:*p* < 0.05—, n.s.—not significant (*p* > 0.05).

The virucidal activity of the NTP-generating devices tested (Laboratory prototypes 1 and 2 and Plasmatico v1.0) was determined using SARS-CoV-2, IAV, HRV and HAdV (Fig. 2, Supplementary Table S1).

After 15 min of NTP exposure, the infectivity of all tested viruses decreased substantially, whereby IAV was the most susceptible virus using Laboratory prototypes 1 and 2 (90 and 88% inactivation, respectively, Fig. 2a, b and 1.0 and 0.9 log_10_ TCID_50_ decrease in corresponding samples, Supplementary Table S1), and HRV when using Plasmatico v1.0 (87% inactivation, Fig. 2c and 1.2 log_10_ TCID_50_ decrease in corresponding sample, Supplementary Table S1). In contrast, SARS-CoV-2 was the most resistant virus using all three devices tested (28–51% inactivation after 15 min and 0.1–0.3 log_10_ TCID_50_ decrease in corresponding samples, Supplementary Table S1). Altogether, after 15 min of NTP exposure, there was no clear trend of a particular device outperforming the others. After 30 min of exposure, all devices inactivated the infectivity of all viruses by approximately 80–90% (about 1 log_10_ decrease in TCID_50_ in corresponding samples, Supplementary Table S1). After 60 min of NTP exposure, all devices abolished HRV infectivity (≥ 99.9% inactivation) and strongly inactivated (≥ 93% and 1.8–4.2 log_10_ TCID_50_ decrease in corresponding samples, Fig. 2, Supplementary Table S1) the other viruses. After 90 min of NTP exposure, Plasmatico v1.0 reduced the TCID_50_ titers of all viruses by more than 3.0 logs_10_ (Supplementary Table S1), which represents 99% reduction of virus infectivity (Fig. 2). In Laboratory Prototypes 1 and 2, complete inactivation of IAV and SARS-CoV-2 required 120 min, while HAdV required 180 min NTP exposure to completely inactivate. The most sensitive to all devices was HRV (complete inactivation with 4.8 log_10_ TCID_50_ decrease with Laboratory prototype 1 after 90 min and complete inactivation after 60 min with 4.7 and 3.3 log_10_ TCID_50_ decrease with Laboratory prototype 2 and Plasmatico v1.0, respectively). While Laboratory Prototypes 1 and 2 achieved complete inactivation of HAdV after 180 min (5.1 and 4.2 log_10_ TCID_50_ decrease, respectively), Plasmatico v1.0 achieved a 4.3 log_10_ TCID_50_ decrease after only 90 min. In conclusion, depending on the virus and device used, 60–180 min of NTP exposure was needed to achieve disinfection of the contaminated parafilm, with Plasmatico v1.0 being the most efficient device tested.

Of note, control virus samples were handled under the same conditions in parallel (without NTP exposure) and used for data normalization in Fig. 2 to reduce bias originating from infectivity reduction due to lengthy incubation, drying and imperfect virus recovery. Indeed, after 60 min of incubation without NTP exposure, infectivity decrease was observed for individual viruses as follows, 0.1 log_10_ TCID_50_ (IAV), 0.3 log_10_ TCID_50_ (SARS-CoV-2), 0.9 log_10_ TCID_50_ (HAdV) and 1.2 log_10_ TCID_50_ (HRV).

### Bactericidal activity of NTP-generating devices against *P. aeruginosa* and MRSA

The bactericidal activity of the NTP-generating devices tested (Laboratory prototypes 1 and 2 and Plasmatico v1.0) was determined by CFU/mL counting of *P. aeruginosa* PAO1 and MRSA ATCC 43300 (clinical isolate) (Table 1). After 15 min of NTP exposure, none of the tested samples reached inhibition allowing for detection of individual colonies on agar plates, thus hampering precise determination of surviving bacteria cells (not quantifiable, n.q.). Strong inhibition of bacterial growth was observed after 30 min NTP exposure in Laboratory prototype 1 for both *P. aeruginosa* and MRSA (24 and 272 surviving CFU/mL, respectively). In the case of *P. aeruginosa*, a complete inhibition (7 log_10_ decrease) was achieved after 60 min of NTP exposure in all three devices tested. MRSA was slightly more resistant, with a few colonies detectable after 60 min, and a complete inhibition (7 log_10_ decrease) after 90 min of NTP exposure in all three devices tested. In conclusion, all three devices exhibit a strong disinfectant effect against the tested bacteria.

**Table 1:** Bactericidal activity of NTP-generating devices (Laboratory prototypes 1 and 2 and Plasmatico v1.0) against *Pseudomonas aeruginosa* PAO1 and methicillin resistant *Staphylococcus aureus* (MRSA) ATCC 43300 (clinical isolate).

NTP exposure time (min)	Laboratory prototype 1	Laboratory prototype 2	Plasmatico v1.0
	CFU/mL	CFU/mL	CFU/mL
*Pseudomonas aeruginosa* PAO1			
0	5 × 10^7^	5 × 10^7^	5 × 10^7^
15	n.q.	n.q.	n.q.
30	24	n.q	n.q
60	0	0	0
90	0	0	0
Methicillin-resistant *Staphylococcus aureus* ATCC 43300 (clinical isolate)			
0	2 × 10^7^	2 × 10^7^	2 × 10^7^
15	n.q.	n.q.	n.q.
30	272	n.q.	n.q.
60	1	3	60
90	0	0	1

Bacteria concentration in control samples was determined using decimal dilution. Results are expressed as colony forming units per milliliter (CFU/mL). All quantifiable results were significant at *p* < 0.05. n.q.—not quantifiable by method used.

### Disinfection of FFP2 masks in Plasmatico v1.0

To further investigate the virucidal and bactericidal efficiency of Plasmatico v1.0, the disinfection of contaminated FFP2 masks was tested (Table 2). In order to unify data presentation for viruses and bacteria, logarithmic decreases are depicted, with bolded numbers indicating complete inactivation of viruses and killing of bacteria. After 60 min of NTP exposure, approximately 50% reduction in infectivity and viability was observed for SARS-CoV-2, IAV, HRV, HAdV, and *P. aeruginosa*, whereas, surprisingly, MRSA viability was inhibited by more than 80%. Complete inactivation of pathogens and hence disinfection of FFP2 masks was achieved after 90 min for all pathogens tested, with the exception of HAdV, which required 120 min.

**Table 2:** Disinfection of FFP2 masks contaminated with viruses (SARS-CoV-2—Severe Acute Respiratory Syndrome Coronavirus 2, IAV—influenza A virus, HRV—human rhinovirus and HAdV—human adenovirus) and bacteria (*Pseudomonas aeruginosa* PAO1 and MRSA—methicillin resistant *Staphylococcus aureus* ATCC 43300—clinical isolate) using Plasmatico v1.0.

Reduction of viral and bacterial contamination of FFP2 masks by treatment in Plasmatico v1.0			
NTP exposure time (min)	SARS-CoV-2 (∆ log_10_)	IAV (∆ log_10_)	HRV (∆ log_10_)
60	2.4	3.6	2.2
90	**5.2**	**6.9**	**3.7**
120	–	–	–

NTP exposure time (min)	HAdV (∆ log_10_)	*P. aeruginosa* PAO1 (∆ log_10_)	MRSA ATCC 43300 (∆ log_10_)
60	2.1	3.7	5.2
90	1.7	**6.5**	**6.4**
120	**4.8**	–	–

Results are expressed in logarithmic decreases of infectious titers/viability. Numbers in bold indicate complete inactivation of viruses and killing of bacteria.

### Evaluation of FFP2 masks material damage inflicted by NTP generated in Plasmatico v1.0

**Fig. 3:**
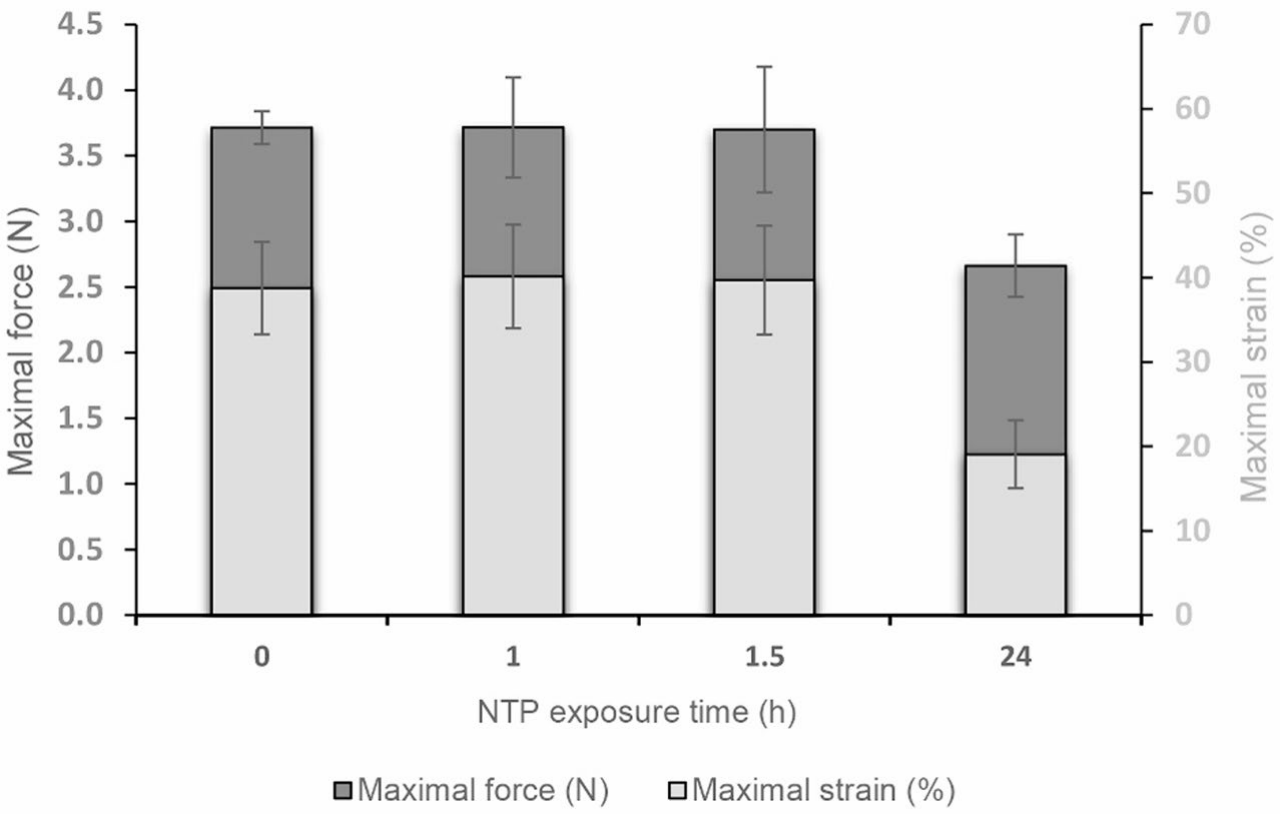
Mechanical properties of FFP2 masks exposed to NTP generated in Plasmatico v1.0. NTP exposure was performed after insertion of virus or bacteria-free FFP2 masks into Plasmatico v1.0 (exposed for 60, 90 min or 24 h). Mechanical properties were analyzed using tensile test. Left y-axis: Maximal force (N), Right y-axis: Maximal strain (%).

To expand on the efficiency data on FFP2 masks disinfection in Plasmatico v1.0, we investigated if damage was inflicted by this disinfection device, using a tensile test to determine the mechanical properties of both reference and NTP-exposed masks (Fig. 3). The results of the tensile tests did not show any substantial changes compared to the reference untreated sample for exposure times of 60 and 90 min. A decrease in maximum force (by approximately 30%, i.e. 1.2 N) and a decrease in maximum strain (by approximately 64%) was detected only after 24 h of exposure. This indicates a decrease in the strength of the fiber structure of the material and a lower ductility (brittleness) of the fibers under tensile deformation.

XPS analysis determines the chemical composition of the tested material surface only to a depth of about 5 nm. The data showed an increase in bound oxygen after exposure to NTP (especially after 24 h) up to 7 at. % (Supplementary Fig. S3), indicating changes taking place on the surface that do not directly translate into changes in material functionality. The increase in oxygen content corresponds to the decrease of C–C; C–H and increase of C–O; C–OH, and to the formation of the carbonyl group (C = O; O–C = O) after 24 h NTP exposure.

On the contrary, SEM analysis (Supplementary Fig. S4) and FTIR spectra (Supplementary Fig. S5) of changes within the FFP2 mask material after exposure to NTP showed no change at all even after the maximum tested exposure time of 24 h (except for the formation of carbonyl group visible in the region around 1730 cm^-1^). These results were also supported by testing the filtration capacity and inhalation and exhalation resistance of the FFP2 masks using paraffin oil aerosol penetration. Quantitative measurements of aerosol penetration showed preservation or even improvement of filtration performance following NTP treatment. The control sample exhibited a baseline pathogen permeability of 2.3446%, while after 90 min of NTP exposure, the permeability decreased to 1.6345%, after 24 h of continuous exposure in the Plasmatico v1.0, permeability was reduced to 0.9846%. This minimal change by 1.36 percentage points after 24 h of treatment remained well below the 6% particle permeability threshold specified by EN 149 for FFP2-class masks. The damage results can be summarized as indicating that there is considerable oxidation on the surface and otherwise the material properties of the FFP2 masks do not change substantially after exposure to NTP.

## Discussion

The enormous impact of the COVID-19 pandemic, which came suddenly and paralyzed the entire world, has highlighted significant weaknesses in our preparedness for such situations^23–25^. In an effort to learn from this pandemic, we focused on one of the major problems, the lack of PPE. We developed a technique for disinfection and hence recycling of PPE, using a DC-based NTP-generating device, preserving its functional properties under relevant EN standards. The reason for using a DC device (although most common commercial devices use AC) is that our DC power supplies are more affordable and have a better safety profile.

NTP is well known for its beneficial properties, such as broad-spectrum inhibitory efficacy against both bacteria and viruses, as demonstrated in our previous studies^9,16,17,22,26,27^, as well as by other researchers^4,28–33^. In two recent studies, we showed that our NTP sources have the ability to disinfect materials sensitive to high temperatures or liquid chemicals without inflicting damage^16,34^. A comparison of the harmfulness of NTP generated by our electrode system with commonly used disinfection methods (UV, chemical, or thermal methods) was presented in our previous study^16^ and shows that NTP is comparable and in some cases more gentle to sensitive materials. This makes NTP a promising tool for disinfecting PPE, often made using nonwoven fabrics sensitive to conventional disinfection methods. Building on these data, we constructed 3D-printed NTP-generating devices (Laboratory Prototype 1 and 2), which were further developed until Plasmatico v1.0 device. We tested these three devices to validate their efficacy against selected important human pathogens (SARS-CoV-2, IAV, HRV, HAdV, *P. aeruginosa* and MRSA) under laboratory conditions (on parafilm carriers and FFP2 masks). As expected, a comparison of the antiviral efficacy of Plasmatico v1.0 in the disinfection of parafilm carriers or FFP2 masks (Fig. 2, Table 2 and Supplementary Table S1) shows that in most cases, the efficiency decreases for FFP2 masks likely due to their complex nonwoven fabric structure as compared to the smooth surface of parafilm. Nevertheless, disinfection of FFP2 masks in Plasmatico v1.0 is definitely possible with a maximum of 30 min delay compared to parafilm. Extended testing of other pathogens, with a particular focus on other multidrug-resistant ESKAPE pathogens, as well as spores, microscopic fungi, and others will be the focus of our next research.

Moreover, we also tested whether the Plasmatico v1.0 inflicted damage to treated materials (in our case the FFP2 masks). Our results showed that Plasmatico v1.0 is highly efficient in inactivating viral and bacterial contaminants on the surface of FFP2 masks (Table 2) without causing substantial changes to the material properties after 90 min of exposure (Fig. 3). Tensile testing, XPS, FTIR analysis and SEM visualization indicated that although increased oxidation was detected at the surface after prolonged exposure to NTP (up to 24 h of exposure), no changes occurred within the material, and they did not substantially affect the overall mechanical integrity of the masks (slight damage started building up after 24 h of exposure without any impact on filtration efficiency). Based on the results obtained, however, it is not possible to determine exactly how many repeated disinfection cycles are still acceptable per FFP2 mask. Although the filtration capacity of the masks does not deteriorate even after 24 h of NTP exposure, and it would therefore be theoretically possible to apply up to 12 disinfection cycles per mask (assuming 120 min per exposure cycle as needed for inactivation of to the most resistant pathogen HAdV), tensile test data indicate slightly deteriorated material properties after 24 h of exposure, and determining the exact number of safe repetitions will therefore be the subject of further research. Altogether, this study provides comprehensive information about the effect of NTP generated in Plasmatico v1.0, i.e., efficiency against relevant human pathogens, and damage to material properties.

Antiviral and antibacterial properties of the NTP generated in our point-to-ring electrode-based devices have already been demonstrated in our small laboratory tubular device comprising a single electrode system^16,17^. However, we needed to achieve a considerable increase in the chamber volume for the insertion of larger items to be disinfected and we therefore attempted to scale-up our devices. Misra et al., 2024 studied the possibilities of increasing the capacity of their NTP-generating device for application in the food industry. The aim of their study was to treat tomatoes on a conveyor belt using a composite system of multiple NTP-generating modules. Similar to our results, their study demonstrated that it is possible to scale-up NTP-generating devices to achieve decontamination or disinfection of larger objects or a larger number of objects. The scale-up of the NTP-generating devices used in our study (Laboratory Prototype 1, 2 and Plasmatico v1.0) showed that by adding a sufficient number of discharge-generating electrode systems, we achieve complete inactivation of pathogens after similar exposure times, even with a considerable increase in the chamber volume. Overall, we were able to achieve the same or even better antiviral efficacy using Plasmatico v1.0 (total inactivation of all tested viruses occurred within 60–90 min, Fig. 2, Supplementary Table S1) when increasing the chamber volume 3.5-fold, from 1560 cm^3^ (Laboratory Prototype 1) to 5440 cm^3^, while quadrupling the number of electrode systems from 2 to 8. A chamber of such volume allows for the insertion of three or more FFP2 masks, thus substantially increasing the efficiency of disinfection with respect to the number of items disinfected at the same time. Therefore, the scale-up of our laboratory prototypes to Plasmatico v1.0 was very successful. Nevertheless, the current exposure times required for disinfection are not practical. Therefore, reduction of exposure times while maintaining efficacy is a primary focus of our future research. Another interesting challenge for future research will be optimizing pathogen inactivation under real-life conditions for use in healthcare or public environments. The possibility of recycling masks used by volunteers both in hospitals and in everyday use, e.g., on public transport, will be tested in this regard.

Many research groups are studying the antiviral or antibacterial properties of NTP and its potential for practical applications, but each of them uses a more or less unique source of NTP. The large number of parameters (e.g., type of discharge, gas used, device power supply, device design) makes the comparison between NTP-generating devices very difficult. NTP-based devices ready for real-life applications very often additionally employ peracetic acid or hydrogen peroxide vapors for disinfection. As discussed by Yardimci and Setlow^35^, such devices cannot be strictly described as “plasma sterilizers”, since their cycles include a purely chemical phase. A simple yet effective device that operates without the addition of gas (most often Ar, Ne) or other chemicals would find many applications in real-world conditions where the supply of additional gases or chemicals may be difficult. To the best of our knowledge, device ready for real life applications with demonstrated antiviral and antibacterial effects, using NTP generated in air atmosphere, is not yet available. Furthermore, only scarce data regarding disinfection of PPE (or FFP2 masks directly) without damage is known. Therefore, Plasmatico v1.0 can be considered very promising for emergency disinfection of PPE, allowing for their reuse.

## Materials and methods

### Non-thermal plasma (NTP)-generating devices

NTP was generated by point-to-ring corona discharge as described in detail in our previous studies^21,22^ with several optimizations to the electrode system and exposure chamber geometry^17,26^. Briefly, the electrode system consists of a point electrode represented by a Medoject 0.6 mm × 25 mm intramuscular injection needle and a ring conical electrode with a diameter of approximately 11 mm at the top and 8 mm at the bottom. The ring electrode was connected to the positive terminal of the high-voltage supply, the point electrode to the negative terminal. The discharge voltage supplied by the source was 7–8 kV and the discharge current was set to 100–150 µA by adjusting the distance between electrodes to approximately 3 mm. In this type of NTP source, the discharge induces an ion wind that carries the active species generated out of the conical electrode.

To scale-up the previously described laboratory tubular device (allowing for disinfection of objects up to 3.5 cm in diameter) for disinfection of face masks or other objects of daily use, while maintaining efficiency, three devices were developed (Fig. 1). Laboratory prototype 1 includes a set of two point-to-ring electrode systems placed at the bottom of a cubic exposure chamber with dimensions of 13 cm × 8 cm × 15 cm. Laboratory prototype 2 includes a set of four point-to-ring electrode systems inside a chamber with dimensions of 13 cm × 16 cm × 15 cm. The latest and commercial prototype, Plasmatico v1.0, contains the largest chamber of 16 cm × 17 cm × 20 cm, allowing for easier handling and user-friendliness. To ensure sufficient disinfection efficiency, Plasmatico v1.0 includes eight sets of discharges, four located at the bottom and four at the top of the chamber.

### Viruses, bacterial strains and cell lines

SARS-CoV-2 (hCoV-19/Czech Republic/NRL_6632_2/2020) was isolated from a nasopharyngeal swab during routine diagnostics in early stages of COVID-19 pandemic in March 2020 in the Czech Republic. Our study used only the propagated virus and did not involve any use of nasopharyngeal swabs from humans. SARS-CoV-2 was propagated in Vero E6 cells (ATCC CRL-1586, obtained from American Type Culture Collection) cultured in Dulbecco’s Modified Eagle’s Medium (DMEM) supplemented with 2% Fetal Bovine Serum (FBS). IAV (H1N1/California/07/2009, Diagnostic Hybrids, Athens, OH, United States) was propagated in MDCK cells (ATCC CCL-34, obtained from American Type Culture Collection) cultured in Influenza growth medium (IGM) supplemented with Pen/Strep, 0.2% Bovine Serum Albumin, 1 mM HEPES, 13.6 mM L-glutamine, 42 mg/L DEAE-Dextran, 1 mg/L TPCK-Trypsin). HRV (species A/type 2, ATCC VR-482) was propagated in Hela Ohio cells (both a kind gift of Heinrich Kowalski) cultured in DMEM with 10% FBS and Pen/Strep. HAdV (species C/type 2, ATCC VR-846) was propagated in A549 cells (DSMZ ACC107) cultured in DMEM with 10% FBS and Pen/Strep.

*P. aeruginosa* PAO1 and MRSA ATCC 43300 (CNCTC 6271, clinical isolate), obtained from the Czech National Collection of Type Cultures, were inoculated on Luria Bertani agar (LB) or blood agar in Petri dishes, respectively.

### Comparison of NTP efficiency at different locations inside device chamber

UV-sterilized parafilm squares (1 × 1 cm) were contaminated with 30 µL of HRV suspension in culture medium displaying a median tissue culture infectious dose of 10^6^ infectious units (IU)/mL, applied in six droplets, each containing 5 µL solution. Droplets were completely dried in a biohazard safety cabinet (40 min) and placed in the following locations (Supplementary Fig. S1) in Laboratory prototype 1: 1—contamination facing the NTP source, directly above the source, 2—contamination facing the NTP source, placed far away from the source, 3—parafilm placed inside a Petri dish, contamination facing away from the NTP source, 4—parafilm glued inside a Petri dish, contamination facing the NTP source. A set of control samples was placed in identical locations in a standby Laboratory prototype 1. In both devices, humidity was increased by adding wet cotton in a Petri dish and NTP was applied for 30 min. Subsequently, residual virus was recovered from the parafilm surface using 200 µL phosphate saline buffer (PBS) and virus titer was determined by a median tissue culture infectious dose (TCID_50_) assay using a 1:10 serial dilution for infecting Hela Ohio cells in 96-well plates. Hela Ohio cells were incubated at 37 °C in a CO_2_ incubator for 5 days, and the cytopathic effect (CPE) was quantified using the Crystal Violet assay, as described previously^36^. Calculation of the respective infectious titers expressed in IU/mL was performed using the Spearman-Kärber method^37,38^.

### Determination of virucidal activity of NTP-generating devices

UV sterilized squares of parafilm (1 × 1 cm) were contaminated with 30 µL of suspension of each virus tested in culture medium displaying a median tissue culture infectious dose ranging from 10^3^ to 10^6^ IU/mL, applied in six droplets, each containing 5 µL solution. Droplets were partially dried in a biohazard safety cabinet (15 min) and contaminated parafilm squares were placed in a Petri dish at the location “3” (contamination facing away from NTP source, Supplementary Fig. S1), previously identified as the most shielded one from NTP exposure. A set of control samples was placed in the biosafety cabinet or another identical device, which was not producing NTP. Humidity was increased by adding ddH_2_O in a Petri dish (6 mL for Laboratory prototypes 1 and 2, 10 mL for Plasmatico v1.0). NTP was applied for different exposure times (ranging from 15 to 180 min) depending on the requirements of device and virus used. Subsequently, residual virus was recovered from the surface of parafilm squares using 200 µL PBS, the material was washed thoroughly (the virus-containing solution was repeatedly re-distributed across the entire contaminated surface). The recovered suspension was used directly for the infection of permissive cells (specified above) to determine the infectious titer of each virus according to the respective protocols described below.

SARS-CoV-2 titer was determined by using 20 µL of each sample diluted into a 24-well plate containing 200 µL of cultivation medium in each well. The viral suspension was further distributed with a dilution step of 1:10. Afterwards, Vero E6 cells (2.5 × 10^5^ per well) were added in 300 µL volume to the suspension and were incubated for 4 h at 37 °C/5% CO_2_. Subsequently, the mixture was overlaid with 500 µL of 3% carboxymethyl cellulose and incubated for 5 days at 37 °C/5% CO_2_. IAV titer was determined by pipetting 20 µL of each sample into a 24-well plate containing 300 µL of IGM in each well. The viral suspension was further distributed with a dilution step of 1:5. MDCK cells (1.8 × 10^5^ per well, plated day before experiment) in a 24-well plate were washed once with PBS, 300 µL of IGM-containing serially diluted sample were added to the cells, and incubated for 1 h at 37 °C/5% CO_2_. After 1 h, cells were washed once with PBS, 300 µL of IGM were added to the cells, and the medium was overlaid with 300 µL of 1.2% carboxymethyl cellulose and let incubated for 2 days at 37 °C/5% CO_2_. For both SARS-CoV-2 and IAV, the infection was terminated by aspirating the medium. Cells were washed once with PBS, and fixed and stained with naphthalene black. After 45 min of incubation, the naphthalene black solution was aspirated, cells were washed with water and the resulting plaques were counted. The viral titer was expressed in plaque forming units (PFU)/mL. HRV was titrated by a TCID_50_ assay using a 1:10 serial dilution for infecting Hela Ohio cells in 96-well plates. Hela Ohio cells were incubated at 37 °C in a CO_2_ incubator for 5 days, and the CPE was quantified using the Crystal Violet assay, as described previously (Lee et al., 2015). Calculation of the respective infectious titers expressed in IU/mL was performed using the Spearman-Kärber method^37,38^. HAdV was titrated by a TCID_50_ assay using a 1:10 serial dilution for infecting A549 cells in 96-well plates. A549 cells were incubated at 37 °C in a CO2 incubator for 7 days, and the CPE was quantified using the Crystal Violet assay, as described previously^36^.

All data were generated in three biological replicates and statistically analyzed using Shapiro–Wilk test (to assess the data distribution). A *p*-value > 0.05 (in most cases very close to 1) indicated that the data follow a normal distribution. A *t*-test (two-sided test comparing treated sample and control sample at given timepoints) was therefore employed to determine the significance of differences. Results displaying *p*-values < 0.05 were considered significant and depicted as average ± standard error of the mean.

### Determination of bactericidal activity of NTP-generating devices

The surface of sterile agar in Petri dishes (LB agar for *P. aeruginosa* and blood agar for MRSA) was inoculated with a bacterial suspension. For disinfection in Laboratory prototypes 1 and 2, agars in Petri dishes (3.5 cm diameter) were inoculated by 50 μL of suspension of approximately 1 × 10^8^ CFU/mL. For disinfection in Plasmatico v1.0, agars in Petri dishes (9 cm diameter) were inoculated by 500 μL of suspension of approximately 1 × 10^7^ CFU/mL. After complete drying in a laminar flow cabinet, Petri dishes were placed in Laboratory prototypes 1 and 2 and Plasmatico v1.0, and exposed to NTP for 15, 30, 60 and 90 min. Due to the presence of agars, the humidity inside the device during treatment is very high (almost 100%), therefore there was no need for an additional humidity source in this case. Exposed Petri dishes containing bacteria were incubated statically overnight at 37 °C. In the case of no or insufficient inhibition of the bacterial layer on the agar, single colonies could not be distinguished, and the number of CFU/mL could not be accurately determined (indicated as "n.q.": not quantifiable by method used, Table 1). Detectable or complete inhibition, resulting in quantifiable colony counts, is indicated in CFU/mL, reflecting residual bacterial concentration. All data were generated in five technical and three biological replicates and statistically analyzed using one-way ANOVA test. Results displaying *p*-values < 0.05 were considered significant and depicted as average ± standard error of the mean.

### Determination of disinfection efficiency of Plasmatico v1.0 using contaminated FFP2 masks

UV-sterilized FFP2 masks were contaminated with 50 µL of each virus suspension tested in culture medium, applied in twenty droplets, each containing 2.5 µL solution. Three contaminated masks were placed simultaneously in Plasmatico v1.0 and exposed to NTP for 60, 90 or 120 min. Humidity during the NTP treatment was increased by adding two Petri dishes with 10 mL of ddH_2_O on the bottom of the device. Subsequently, residual virus was recovered from the surface of FFP2 masks using 450 µL culture medium, the material was washed thoroughly, the virus-containing solution was repeatedly re-distributed across the entire contaminated surface. Evaluation of the residual virus infectivity was performed using the same procedures as described above. For experiments with bacterial strains, contamination of FFP2 masks was performed as for viruses with just a few slight differences. The exposure times were 60 and 90 min, and the washing was done using 450 µL of PBS. The evaluation of the bactericidal effect was performed as described above, but a decimal dilution of samples was employed to increase assay sensitivity, hence the CFU/mL of surviving bacteria could be determined for all exposure times tested. Results were expressed as a logarithmic decrease of CFU/mL.

### Determination of FFP2 masks material properties exposed to NTP in Plasmatico v1.0

The following material tests were applied to FFP2 masks exposed to NTP for 60 and 90 min (corresponding to the time of disinfection cycle needed for inactivation of most of the tested pathogens) and for 24 h (testing intense exposure corresponding to the time of 12 repeated disinfection cycles).

### Mechanical properties determination using tensile test

The tensile test is one of the most widely used static methods for measuring mechanical properties. Samples were taken from the inside of FFP2 masks (cotton-like form) and cut out in the shape of 5A dog bone. The tensile test was measured using a ZWICK 010 (Germany) device with a 10 N load cell with the following parameters: initial grip-to-grip distance: 50 mm, preload speed 1 mm/min to 0.1 N, test speed 10 mm/min, end of test: decrease of 80% of F_max_. The parameters of maximum force related to material strength and maximum strain related to material ductility under mechanical loading were evaluated.

### Chemical composition analysis of material surface using X-ray photoelectron spectroscopy (XPS)

X-ray photoelectron spectroscopy (XPS) analyses, which monitors the chemical composition of the sample tested to a depth of approximately 5 nm, were performed on an Axis Ultra DLD spectrometer (Kratos Analytical Ltd, UK) using a monochromatic Al Kα (hν = 1486.7 eV) X-ray source operating at 75 W (5 mA, 15 kV). Spectra were obtained using an analytical area of approximately 300 × 700 µm. The high-resolution spectra were measured with a step size of 0.1 eV and pass energy 20 eV. The Kratos charge neutralizer system was used for all analyses. The instrument base pressure during the measurements was consistently at 2 × 10^−8^ Pa. Spectra were analyzed using CasaXPS software (version 2.3.17) and were charge-corrected to the main line of the spectral carbon C 1 s component (C–C, C–H) set to 285.00 eV. The standard Shirley background was used for all sample spectra.

### Chemical structure analysis using Fourier transform infrared spectrophotometry (FTIR)

Fourier transform infrared spectrophotometry (FTIR) was performed on a Bruker Tensor 27 device in the attenuated total reflection mode in the spectral area 4000–600 cm^−1^ with a resolution of 4 cm^−1^ and a number of scans 32. For samples with hydroxyl- and epoxy- functions, diamond crystal was used due to its low refractive index and high hardness. Since diamond has an absorbance in the region 2200–1800 cm^−1^, germanium crystal was used for samples with nitrogen reagents.

### Microscopic morphology visualization using scanning electron microscopy (SEM)

The surface microstructure and morphology of FFP2 masks were visualized using scanning electron microscope Vega II LSH (TESCAN Orsay Holding, a.s., Brno, Czech Republic) with a maximal resolution of up to 3 nm at an accelerating voltage of 30 kV. Cut samples of approximately 5 × 5 mm were placed on an aluminum stage and sputtered with gold in an argon atmosphere using a Sputter Coater POLARON SC7640 (Newhaven, United Kingdom of Great Britain and Northern Ireland, 10 mA, 60 s) before measurement. The applied accelerating voltages (HV) in kV, working distances in mm (WD), probe currents (PC) and magnifications are presented in the bottom part of the result figures.

### Filtration efficiency and breathing resistance analysis using paraffin oil aerosol penetration

The penetration of FFP2 masks (3 M Aura 9320 +) untreated and treated 24 h with NTP in Plasmatico v1.0 was measured using an aerosol generator and photometric measuring system (Lorenz Meβgerätebau FMP 03) with a differential pressure sensor, as described previously^16^. The device was certified as a test system according to the following standards: EN 143 (Respiratory protective devices—Particle filters—Requirements, testing, marking), and EN 149 (Respiratory protective devices—Filtering half masks to protect against particles—Requirements, testing, marking). Each mask was positioned in the test system, where aerosolized paraffin oil was injected on one side, and the penetration through the mask was measured photometrically on the opposite side. Simultaneously, breathing resistance was measured by maintaining a constant airflow on one side of the mask while recording pressure differentials on the opposite side using the differential pressure sensor. Paraffin oil was selected over NaCl aerosol to better simulate liquid aerosols, including biological aerosols, ensuring a more stringent evaluation aligned with higher filtration standards (e.g., P3 filters).The aerosol generator produced a defined amount (6 mg/3 min ± 0.2 mg) of aerosolized paraffin oil, the test system passed it through the material, and the photometer situated on the other side of the sample measured the aerosol concentration, thereby indicating the retention efficiency (filter penetration). The particle size distribution was approximately 0.1–2 μm (geometric mean 0.44 μm), which is close to the most frequently observed penetrating particle size. The output of the aerosol generator was set to 150% with a flow of 95 L/min, an atomizer pressure of 5 bar and an oil temperature of 60 °C. The test was performed for 3 min.

## Conclusion

In conclusion, this study demonstrates the successful development and provides evaluation of the Plasmatico v1.0 device equipped with multi-point electrode systems, which generates NTP for the effective disinfection of everyday items, including FFP2 masks. All prototypes used during the development of this scaled-up device showed high virucidal and bactericidal activity, with Plasmatico v1.0 achieving complete inactivation of SARS-CoV-2, IAV, HRV, HAdV, *P. aeruginosa*, and MRSA within 90–120 min of exposure. Material properties tests on NTP-treated FFP2 masks revealed no deterioration in material properties nor filtration performance after 90 min. Surface oxidation and slight damage to mechanical properties were observed after 24 h treatment, but this did not compromise the integrity of the mask, and filtration capacity remained sufficient in accordance with the EN 149 standard for FFP2 masks. These results confirm that Plasmatico v1.0 is a scalable, effective, and material-safe disinfection technology that supports the wider adoption of NTP for applications in healthcare, personal protection, and public hygiene. Future work will focus on reducing the required exposure time, determining the exact number of acceptable disinfection cycle repetitions, and expanding implementation to different types of materials and applications.

## Supplementary Information

Below is the link to the electronic supplementary material.

Supplementary Material 1

## Data Availability

Data is provided within the manuscript or supplementary material.
