# Supplementary material for: Scaled-up non-thermal plasma-generating device Plasmatico enables effective and harmless disinfection of personal protective equipment
Source: Sci Rep. 2025 Oct 13;15:35631. doi: 10.1038/s41598-025-19520-7 (PMC12518771; doi:10.1038/s41598-025-19520-7)
Supplement: Supplementary file 1 — Supplementary Material 1 [file 41598_2025_19520_MOESM1_ESM.docx]

**SUPPLEMENTARY INFORMATION**


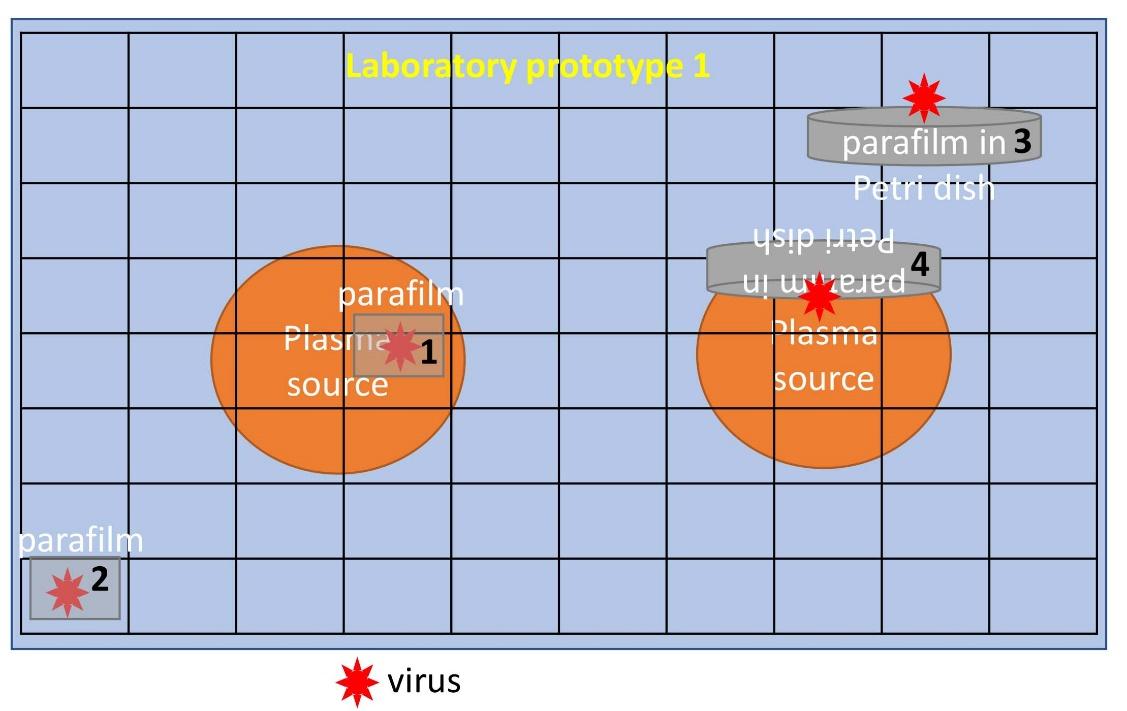


**Supplementary Figure S1** Schematic of sample locations inside Laboratory prototype 1 used to identify position with lowest NTP efficiency. Electrode systems generating non-thermal plasma (NTP) source are located at the bottom of the chamber. 1 - contamination facing down, directly above the source, 2 - contamination facing down, placed away from the source, 3 - parafilm placed inside a Petri dish, contamination facing up, 4 - parafilm glued inside a Petri dish, contamination facing down.


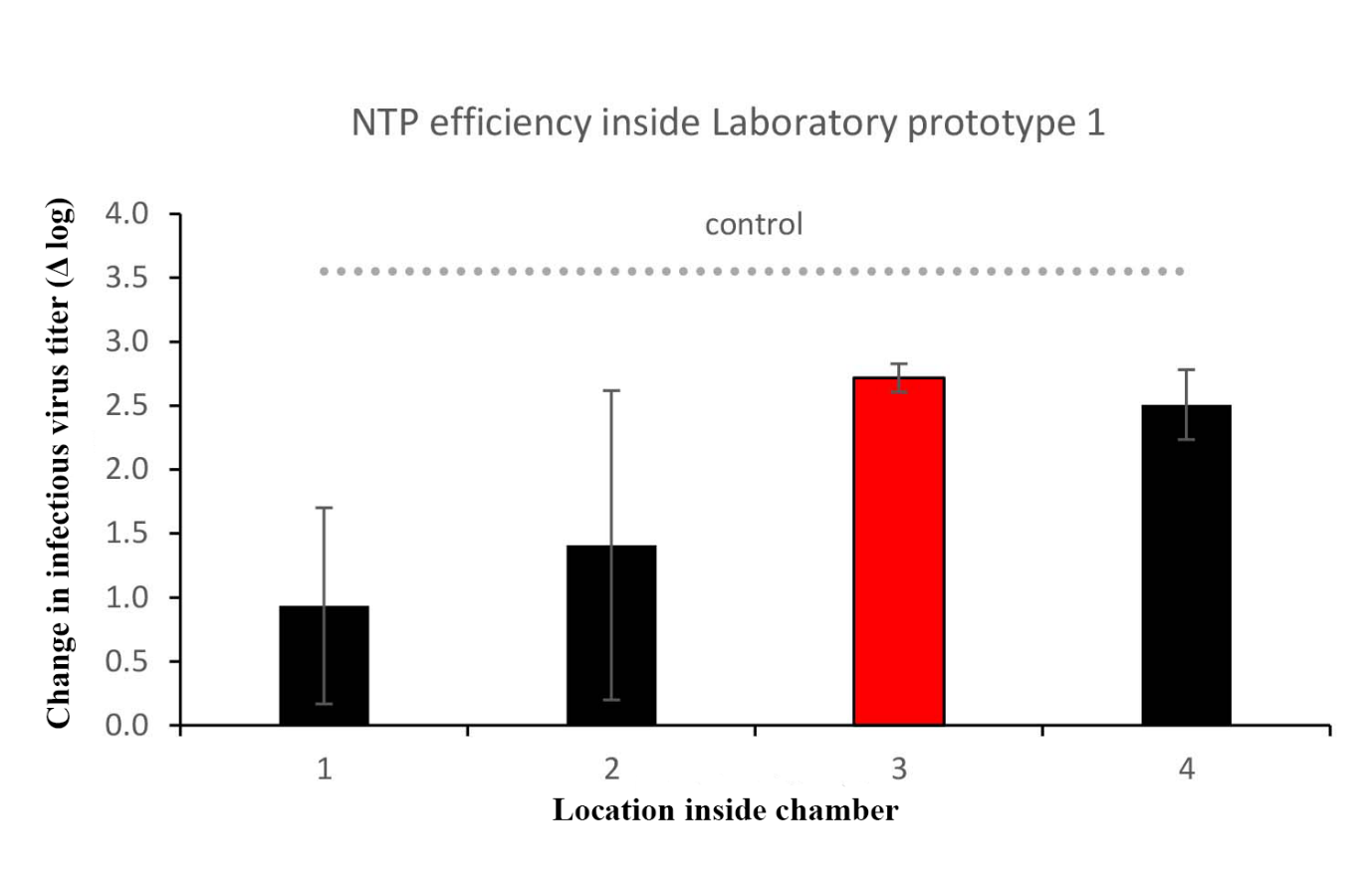


**Supplementary Figure S2** Virucidal effect was lower in samples facing away from NTP source. 1 -contamination facing the NTP source, directly above the source, 2 - contamination facing the NTP source, placed far away from the source, 3 - parafilm placed inside a Petri dish, contamination facing away from the NTP source, 4 - parafilm glued inside a Petri dish, contamination facing the NTP source. Locations with human rhinovirus (HRV) contamination facing the NTP source (1, 2 and 4) yielded more efficient disinfection than the one facing away (3) after 30 min of NTP application in Laboratory prototype 1. The least efficient placement (highlighted in red) was selected for large-scale testing in all three devices tested in the current study.

*
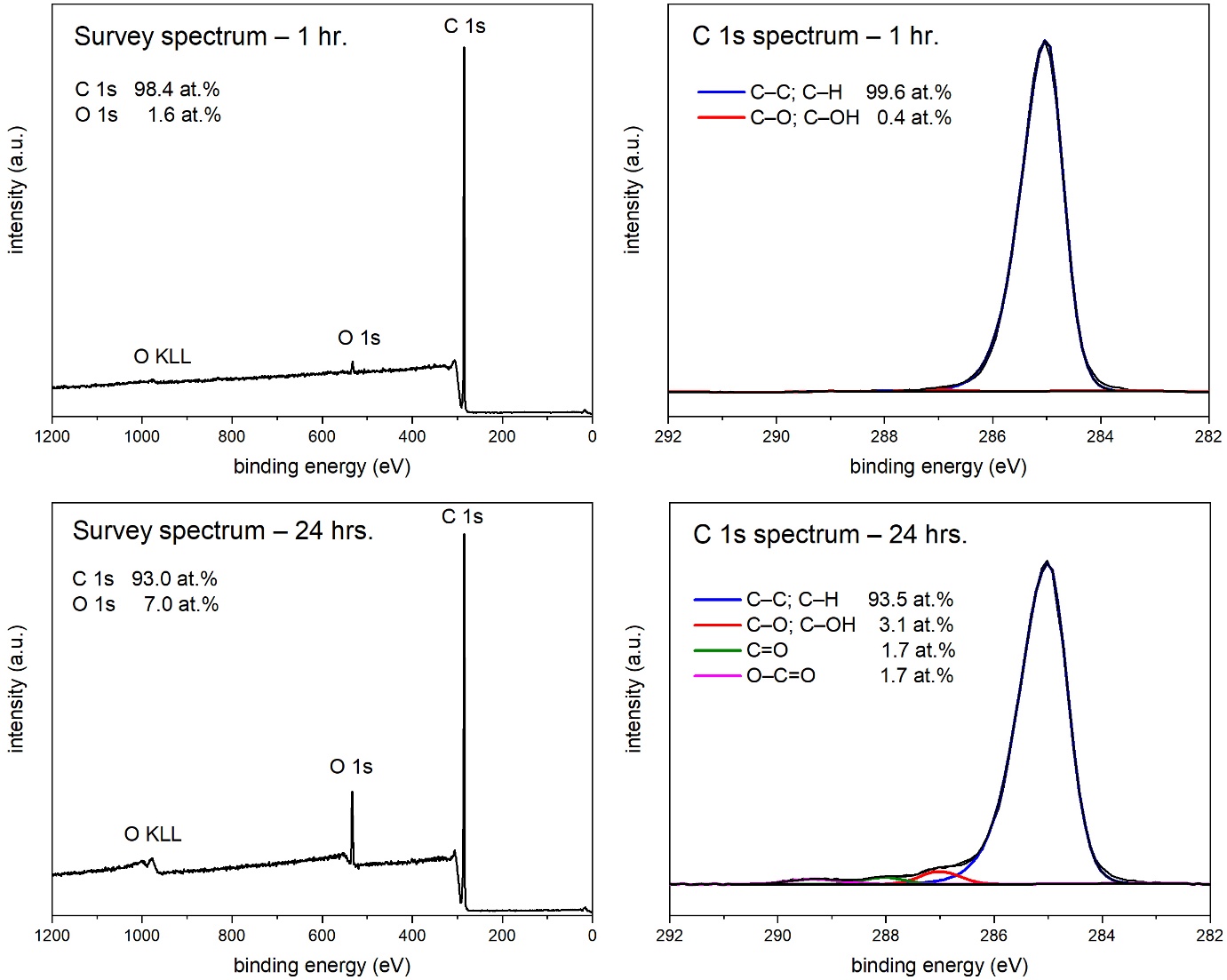
*

**Supplementary Figure S3** XPS survey spectra of FFP2 masks exposed to non-thermal plasma (NTP) in Plasmatico v1.0 for 1 h (top) or 24 h (bottom).


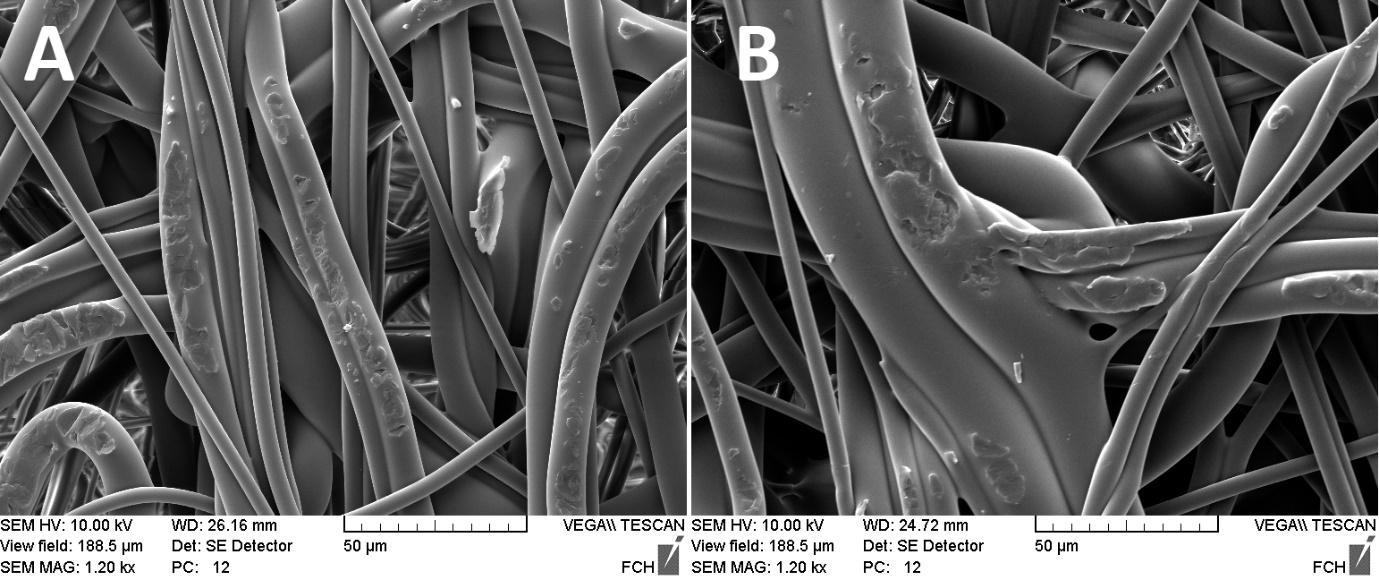


**Supplementary Figure S4** Scanning electron microscopy (SEM) images of FFP2 masks exposed to non-thermal plasma (NTP) for 24 h in Plasmatico v1.0. A – untreated FFP2 mask, B – 24 h NTP-exposed FFP2 mask. Scale bar = 50 µm.


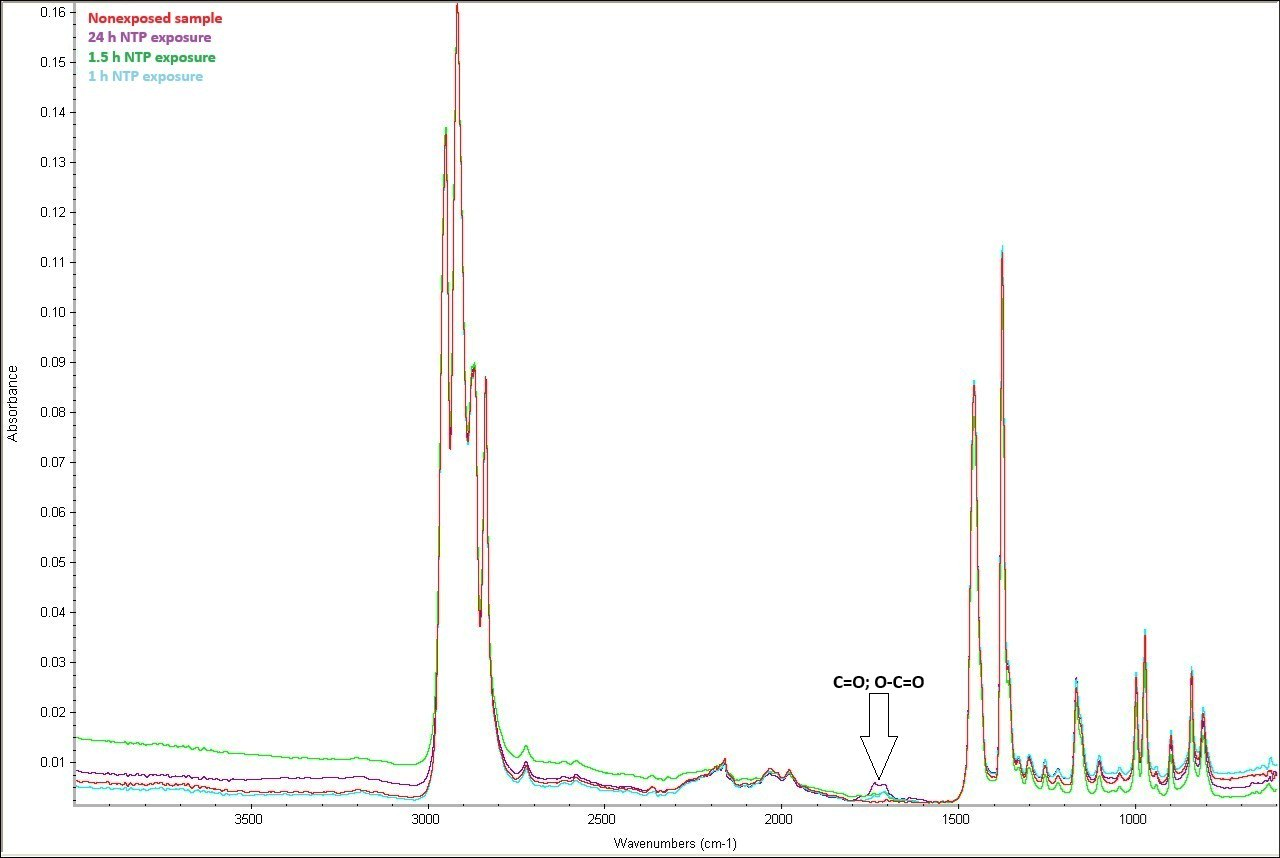


**Supplementary Figure S5** FTIR spectra of FFP2 masks exposed to non-thermal plasma (NTP) in Plasmatico v1.0. Untreated sample (red); 1 h NTP exposure (blue); 1.5 h NTP exposure (green); 24 h NTP exposure (purple).

**Supplementary Table S1** Virucidal activity of three NTP-generating devices tested (Laboratory prototypes 1 and 2 and Plasmatico v1.0) using Severe Acute Respiratory Syndrome Coronavirus 2 (SARS-CoV-2), influenza A virus (IAV), human rhinovirus (HRV) and human adenovirus (HAdV). NTP exposure was performed after contamination of parafilm squares by virus suspensions and their insertion into each device. Results are expressed as logarithmic decreases of infectious virus titers. Complete inactivation is highlighted in bold.

| SARS-CoV-2 (∆ log infectious titer) | | | | | | | | | | | | | | | | | | | |
| --- | --- | --- | --- | --- | --- | --- | --- | --- | --- | --- | --- | --- | --- | --- | --- | --- | --- | --- | --- |
| Laboratory prototype 1 | | | | | |  | Laboratory prototype 2 | | | | | |  | Plasmatico v1.0 | | | | | |
| NTP exposure time (min) | | | | | |  | NTP exposure time (min) | | | | | |  | NTP exposure time (min) | | | | | |
| 15 | 30 | 60 | 90 | 120 | 180 |  | 15 | 30 | 60 | 90 | 120 | 180 |  | 15 | 30 | 60 | 90 | 120 |  |
| 0.3 | 1.0 | 2.1 | 3.1 | **6.0** | - |  | 0.1 | 0.9 | 2.0 | 3.1 | **6.0** | - |  | 0.3 | 1.2 | 1.8 | 3.1 | **5.1** |  |
| IAV (∆ log infectious titer) | | | | | | | | | | | | | | | | | | | |
| Laboratory prototype 1 | | | | | |  | Laboratory prototype 2 | | | | | |  | Plasmatico v1.0 | | | | | |
| NTP exposure time (min) | | | | | |  | NTP exposure time (min) | | | | | |  | NTP exposure time (min) | | | | | |
| 15 | 30 | 60 | 90 | 120 | 180 |  | 15 | 30 | 60 | 90 | 120 | 180 |  | 15 | 30 | 60 | 90 | 120 |  |
| 1.0 | 1.0 | 2.6 | 3.7 | **4.7** | - |  | 0.9 | 1.0 | 2.6 | 3.4 | **4.2** | - |  | 0.6 | 0.8 | 6.2 | **6.2** | - |  |
| HRV (∆ log infectious titer) | | | | | | | | | | | | | | | | | | | |
| Laboratory prototype 1 | | | | | |  | Laboratory prototype 2 | | | | | |  | Plasmatico v1.0 | | | | | |
| NTP exposure time (min) | | | | | |  | NTP exposure time (min) | | | | | |  | NTP exposure time (min) | | | | | |
| 15 | 30 | 60 | 90 | 120 | 180 |  | 15 | 30 | 60 | 90 | 120 | 180 |  | 15 | 30 | 60 | 90 | 120 |  |
| 0.7 | 1.4 | 3.2 | **4.8** | **4.8** | - |  | 0.2 | 1.1 | **4.7** | **4.7** | - | - |  | 1.2 | 1.4 | **3.3** | **3.3** | - |  |
| HAdV (∆ log infectious titer) | | | | | | | | | | | | | | | | | | | |
| Laboratory prototype 1 | | | | | |  | Laboratory prototype 2 | | | | | |  | Plasmatico v1.0 | | | | | |
| NTP exposure time (min) | | | | | |  | NTP exposure time (min) | | | | | |  | NTP exposure time (min) | | | | | |
| 15 | 30 | 60 | 90 | 120 | 180 |  | 15 | 30 | 60 | 90 | 120 | 180 |  | 15 | 30 | 60 | 90 | 120 |  |
| 1.0 | 1.3 | 2.0 | 2.2 | 2.6 | **5.1** |  | 2.1 | 2.6 | 4.2 | 3.8 | 3.4 | **4.2** |  | 1.2 | 1.6 | 3.8 | **4.3** | **4.3** |  |
